# Prevalence and Predictors of Obesity among 7- to 17-Year-Old Schoolchildren in Urban Arusha, Tanzania

**DOI:** 10.1155/2019/3106597

**Published:** 2019-10-29

**Authors:** Haji Chomba, Haikael D. Martin, Judith Kimywe

**Affiliations:** ^1^Department of Food Biotechnology and Nutrition Sciences, Nelson Mandela African Institution of Science and Technology, P. O. Box 447, Arusha, Tanzania; ^2^Department of Foods, Nutrition and Dietetics, Kenyatta University, Box 43844-00100, Nairobi, Kenya

## Abstract

**Background:**

Childhood obesity is currently increasing at an alarming rate worldwide. Childhood obesity research has not been reported in urban Arusha before. This is therefore the first study to investigate the prevalence and predictors of childhood obesity in urban Arusha.

**Methods:**

A cross-sectional study was conducted involving 451 schoolchildren. Overweight was defined to range from 85^th^ to 94^th^ BMI percentile for age and sex while obesity was defined as above 94^th^ BMI percentile for age and sex. Chi-square test was used for comparison between child sex and sociobehaviors, and multiple logistic regression was used to determine the significant predictor factors at *P* values = 0.05.

**Results:**

The overall prevalence of overweight and obesity was 17.7% (80/451) with 12.6% (57/451) being obese and 5.1% (23/451) being overweight. Results from univariate logistic regression showed child sex, random sleeping time, and random eating habit were the significant predictor factors. However, when all the predictor factors were used in the final multiple logistic regression model, only random sleeping time and random eating habit of different food items irrespective of their nature were significant at *P*=0.000, AOR = 4.47, and 95% CI = 2.00–10.01, and *P*=0.012, AOR = 2.54, and 95% CI = 1.23–5.33, respectively.

**Conclusions:**

The prevalence of obesity was as higher as twice the prevalence observed in other previous studies in Tanzania. Being a girl, random sleeping time and random eating habit were independent predictors. In addition to larger sample sizes, longitudinal studies are needed in order to track individuals and population level trends in BMI over time.

## 1. Introduction

Prevalence of overweight and obesity is on rise worldwide [1, 2] and recently has become a serious global public health concern [3, 4]. Besides, obesity affects all sexes, ages, races, socioeconomic groups, and ethnicities [5–8]. Furthermore, it has been reported as a major risk factor for type 2 diabetes, cardiovascular disease, hypertension, stroke, and certain forms of cancer [9–11]. Likewise, childhood obesity is currently increasing at an alarming rate worldwide [12, 13] regardless of the existing efforts for its management and prevention [14]. Several factors have been reported as drivers for both global and childhood obesity. These include rapid economic growth, rapid urbanization, reduced physical activity, and overconsumption of cheap, palatable, more energy-dense, nutrient poor foods with high levels of sugar and saturated fats [15–18].

Tanzania is one among African countries currently experiencing the rapid economic growth, urbanization, and nutrition transition [19–21]. Therefore, it is apparent that Tanzania is not exempted from the epidemic of childhood obesity [22–26]. However, data on childhood obesity are pertinent in Tanzania. To date, there is no published research study on childhood obesity in urban Arusha. Therefore, this study will shed light on the extent of the problem among 7- to 17-year-old schoolchildren.

## 2. Methods

### 2.1. Study Setting

This study was conducted in urban Arusha City between May and August, 2018. Arusha City is located in the northeastern part of Tanzania and is an important tourism centre for regional and continental activities. It lies below the equator between latitudes 2° and 6°, and longitudinally, the region is situated between 35° and 38° east of Greenwich [27].

### 2.2. Sample Size Determination

The sample size was calculated using Kish and Lisle formula for cross-sectional studies considering proportion of overweight of 40% by Damian et al. [28], 95% confidence interval (CI), and 5% margin of error with 25% for nonresponse rate. Finally, a total of 500 participants were obtained.

### 2.3. Sampling Procedures

A sampling frame of 151 primary schools within Arusha City was used to obtain a total of 12 representative schools. First, all schools were grouped into three divisions, and then from each division, schools were grouped into four strata. Strata were formed based on school ownership such as public or private school and location such as central urban location and periurban location. The four strata were as follows: private urban owned schools, government urban owned schools, private periurban owned schools, and government periurban owned schools. Finally, a simple random sampling technique was applied to select one school from each stratum, hence making a total of 4 schools per division. However, only eight schools, six governments owned and two private owned, were included in the study leaving out four schools that did not consent to participate. Within each selected school, one class was randomly selected and all children from the selected class were invited to participate in the study.

### 2.4. Anthropometric Measurements

Anthropometric measurements were taken by trained nurses early in the morning at the respective schools. Measurements were conducted in a dedicated room at each school with children wearing light clothes and with no shoes. Body weight was measured using a self-calibrating precision digital scale (Omron, Japan). Height was measured to the nearest 0.1 cm by stadiometer with the Frankfort plane in the horizontal position. BMI was calculated as weight in kilograms divided by height in meters squared. BMI percentile was determined using WHO Child Growth Standards in which overweight was defined to range from 85^th^ to 94^th^ BMI percentile for age and sex while the overall obesity was defined as above 94^th^ BMI percentile for age and sex [29].

### 2.5. Sociodemographic Characteristics

The sociodemographic questionnaire (SDQ) was used to collect information on the children's socioeconomic background, physical activity, and sedentary lifestyle (daytime sleeping, playing computer and video games, staying idle at home, and not engaging in household duties like cleaning and washing clothes). In order to get valid information, questionnaires for children below 12 years old were completed by parents/care givers. The questionnaires were developed in both English and Swahili languages for easy comprehension to all pupils as only these two languages were used for instruction at those schools. Information, such as sleep timing hours, sports and programmed exercise performed in a week, time spent daily watching TV, time spent daily at school, and the kind and duration of games usually played, was included in the questionnaires. For sleeping time, four categories were defined as follows: the first category (normal) included participants reported to go to bed in between 8:00 pm and 10:00 pm, second category (late) included participants reported to go to bed in between 10:00 pm and 11: 00 pm, the third category (too late) included participants reported to go to bed in between 11:00 pm and 12:00 pm, and the last category included participants reported to go to bed any time in between 8:00 pm and 12:00 pm. To be able to classify participants into random categories, participants were required to mention more than 3 different times such as 8:00, 9:00, 10:00, 12:00 pm when they went to bed during the week.

### 2.6. Eating Habits

Food frequency questionnaire (FFQ) was used to obtain dietary history on the type and frequency of foods consumed by the respondents for 7 days. Various foods from different food groups and information regarding snack type, preference, consumption frequency, and place of consumption were also included. Participants were grouped into four categories based on the frequency of consumption and type of food items consumed. These were defined as follows. Ate fried foods included participants reported to eat at school food stuffs such as buns, chapatti, crisps, pie, and pan cakes for at least 5 days per week. Ate refined boiled maize and rice included participants reported to eat at school rice and a local food called *makande* made up of beans and dehulled maize for at least 5 days per week. Ate any food included participants reported to eat at school different food categories such as *makande*, pan cake, crisps, juice, and among others randomly without a particular trend of eating a particular food consecutively for at least 5 days per week. Ate food containing more sugars included participants who reported to eat at school food stuffs such as cakes, soda, juices, biscuits and chocolate, and ate nothing included participants reported not to eat anything when were at school until they went homes. Parents whose children were below 12 years were guided to fill in the questionnaires by the trained nutritionists accompanied the researcher. Only meals, snacks, and frequency of eating were the targets. Portion sizes were not calculated since only eating habits and food preferences were required for this study.

### 2.7. Validity and Reliability

Content validity and relevance of the used questionnaires were tested by experts in the field of overweight and obesity researches from Kenyatta University, Nairobi, Kenya, and Nelson Mandela African Institution of Science and Technology (NM-AIST), Arusha, Tanzania. In addition, the questionnaires were pretested for accuracy and clarity in one of the schools with similar characteristics to the participating schools. Suggestions and shortcomings observed during pilot study were incorporated, and finally, a comprehensive questionnaire for the study was finalized.

### 2.8. Ethical Considerations

Ethical clearance was obtained from National Institute of Medical Research (NIMR) of Tanzania. The permits to visit schools were sought from Arusha City council director. Prior to data collection, parents/caregivers on invitation by the headmasters/headmistresses were informed and briefed about the objectives, significances, and benefits of the study. Parents/caregivers, who agreed to participate, were given the consent forms to sign and thereafter were given two questionnaires one at a time and instructed on how to fill in. Children were included only if parents/caregivers signed the informed consent form. Verbal assent was also obtained from each child before participation.

### 2.9. Statistical Analysis

All statistical analyses were performed using R statistical package version 3.5.2. Continuous variables were summarized by means and standard deviations. Categorical variables were summarized by frequencies and percentages. Chi-square test was used for comparison between child sex and sociobehaviors. Multiple logistic regression was used to determine the significant predictor factors at *P* value = 0.05. First, the univariate logistic regression was performed and explanatory variables with *P* value less than 0.2 were included into stepwise multiple logistic regression models. Akaike information criterion: AIC=2*k* − 2 log *L*=2*k*+deviance (where *k* = number of parameters), was used to derive the final best-fit model.

## 3. Results

About 500 children and their parents/caregivers were invited to participate in the study, but only 451 children and 168 parents/caregivers consented and hence a response rate of 90.2% (451/500) and 33.6% (168/500), respectively. Out of 451 children 58% (260/451) were girls and 42% (191/451) were boys (Table 1). Out of 64 parents reported to be in low-socio-income class, 65.6% were girls' parents and 34.4 were boys' parents. For the parents reported to engage in entrepreneurship, 70% were girls' parents and 30% were boys' parents.

In addition, 41.3% (50/121) of girls' parents and 58.7% (71/121) of boys' parents had attained primary education and 36% (9/25) of girls' parents and 64% (16/25) of boys' parents had secondary education (Table 1). The overall participants' mean age was 12.5 (1.4) of which 12.4 (1.3) and 12.7 (1.4) were the mean ages for girls and boys, respectively (Table 2).

Out of 264 participants reported to wake up in between 5:00 am and 6:00 am, 64.8% were girls and 35.2% were boys, and out of 42 participants reported to wake up in between 6:00 am and 7:00 am, 35.7% were girls and 64.3% were boys. Out of 26 participants reported to wake up in between 5:00 am to 8:00 am, 69.3% were girls and 30.7% were boys. Among the very few reported to wake up in between 7:00 am and 8:00 am, 75% were girls and 25% were boys. Out of 237 participants reported to go school on foot, 61.2% were girls and 38.8% were boys. Out of 99 participants reported to go school by bus, 60.6% were girls and 39.4% were boys. Out of 53 participants reported high level of sedentary behavior (TV watching), 47.2% were girls and 52.8% were boys. Out of 117 participants reported in between 2 and 3 hrs in TV watching, 65% were girls and 35% were boys, and out of 117 participants reported less than 2 hrs in TV watching, 58.1% were girls and 41.9% were boys. Out of 49 participants reported no TV watching, 73.5% were girls and 26.5% were boys (Table 3).

Majority of participants (86% (288/335)) reported to consume snacks; however, the tendency of eating highly processed snacks and sugar-coated snacks among the participants was low. About 66% (221/335) of participants did not skip any meal per day while 17% (58/335) reported to skip breakfast and 10% (36/335) reported to skip lunch. Half of the participants (50.6% (170/335)) reported to eat all meals at home and the rest (49.4%) reported to eat either at home or away from home. More than half of the participants (50.7% (170/335)) used to eat lunch meal at home while 49.3% (165/335) reported to have lunch at school. Different food preferences were reported by the participants such that 36.9% (124/335) reported to eat fried foods, 33.9% (114/335) reported to eat dehulled boiled maize or rice, and 24.1% (81/335) reported random eating of different food categories such as *makande*, pan cake, crisps, juice, and among others without a particular trend of eating a particular food consecutively for at least 5 days per week; in this category, food products containing more saturated fats, sugar, and salts were preferred compared to healthy foods and 3.5% (12/335) reported to prefer sugar-containing foods (Table 4).

**Figure 1:**
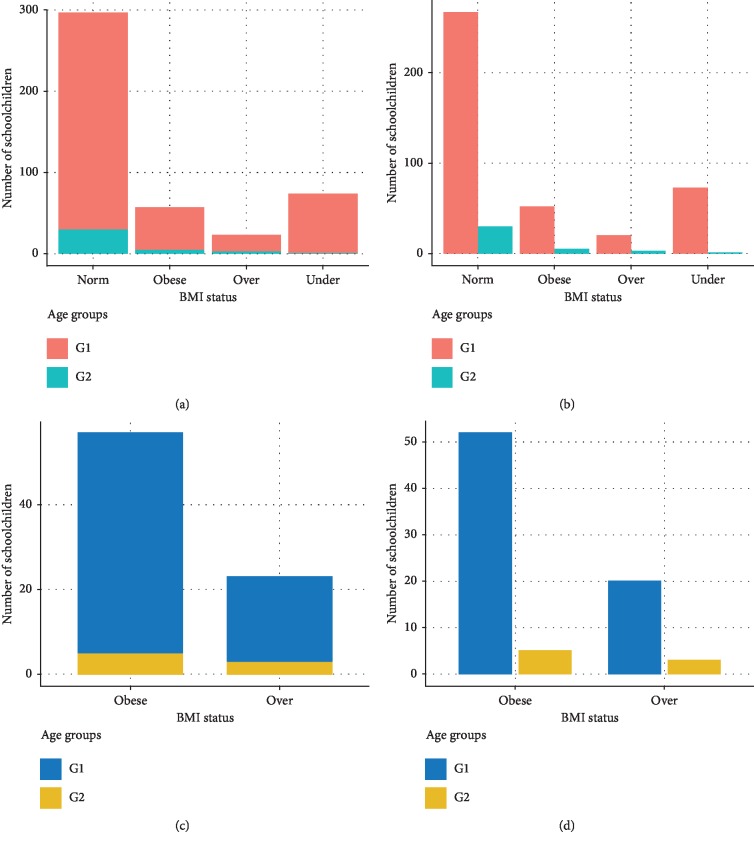
(a) Stacked bar graph for the BMI status of study population (schoolchildren) by age groups (age <11 is G1 and age >10 is G2). (b) Grouped bar graph for the BMI status of study population (schoolchildren) by age groups (age <11 is G1 and age >10 is G2). (c) Stacked bar graph for the prevalence of overweight and obesity of study population (schoolchildren) by age groups (age <11 is G1 and age >10 is G2). (d) Grouped bar graph for the prevalence of overweight and obesity of study population (school children) by age groups (age <11 is G1 and age >10 is G2).

The overall prevalence of overweight and obesity was 17.7% (80/451) with 12.6% (57/451) being obese and 5.1% being overweight (Figures 1(a)–1(d)). However, 14.6% (38/260) of girls were obese and 9.9% (19/191) of boys were obese (Table 5). Children (49.6% (166/335)) involved in school sports less than 2 days per week had higher level of obesity (15.7%) and overweight (4.2%) than both children (37.3% (125/335)) got involved 2 to 4 days per week and children (4.2% (14/335)) got involved more than 4 days per week. Out of 117 children reported to go sleep at night in between 10:00 pm and 11:00 pm (late), 15% (18/117) were obese, out of 169 children reported to go sleep in between 8:00 pm and 10:00 pm (normal), 9.5% (16/117) were obese, and out of 45 children reported to go sleep in between 8:00 pm and 12:00 pm (random), 26.7% (12/45) were obese.

Children reported to wake up in between 5:00 am and 6:00 am after overnight sleep had higher prevalence for both overweight (5.3% (14/263)) and obesity (13.7% (36/263)) followed by the prevalence of obesity (26.9% (7/26)) and overweight (3.8% (1/26)) for children reported to wake up in between 5:00 am and 8:00 am (Table 5).

Children reported random eating of different food categories such as *makande*, pan cake, crisps, juice, and among others without a particular trend of eating a particular food consecutively for at least 5 days per week at school had high prevalence for obesity 22.2% (18/81) and overweight 9.9% (8/81) followed with children preferred fried foods. Children reported to eat lunch every day at home had higher prevalence for both overweight (4.7% (9/190)) and obesity (13.2% (25/190)) than children used to get lunch either at home or at school (Table 6).

### 3.1. Potential Factors for Overweight and Obesity

Predictor factors for overweight and obesity assessed under this study were age, child sex, sedentary habit (TV watching), physical activity, school dietary preferences, meal frequency, eating places, lunch eating places, sleeping time, wake-up timing, and parental sociodemographic factors. Results from univariate logistic regression showed child girl sex, random sleeping time, and random eating of different food categories such as *makande*, pan cake, crisps, juice, and among others without a particular trend of eating a particular food consecutively for at least 5 days per week at school were the significant predictor factors (Tables 5 and 6).

However, when all predictor factors were used in the final multiple logistic regression model, only random sleeping time and random eating of different food categories such as *makande*, pan cake, crisps, juice, and among others without a particular trend of eating a particular food consecutively for at least 5 days per week at school were significant at *P*=0.000, AOR = 4.47, and 95% CI = 2.00–10.01, and *P*=0.012, AOR = 2.54, and 95% CI = 1.23–5.33, respectively (Table 7).

## 4. Discussion

The overall prevalence of overweight and obesity was 17.7% with 12.6% being obese and 5.1% being overweight. The prevalence of obesity was as higher as twice the prevalence observed in other previous studies in Tanzania [30–32]. However, similar or high prevalence has recently been reported in other countries as well [33, 34]. Under univariate logistic regression results, child sex girl was an independent predictor factor for overweight and obesity. Girls were at higher risk of becoming overweight or obese than boys at *P* values = 0.021, OR = 2.06, 95% CI = 1.11–3.82. The same observations have been reported by other studies as well [35–37]. This can partly be explained by the differences in activeness and sedentary behaviors (TV watching) between girls and boys observed in this study. However, biological differences between girls and boys especially during puberty and adolescence period cannot be underestimated as a confounding factor to this difference [38–40].

Though physical activity and sedentary behaviors were not statistically significant, children reported involvement in school sports less than 2 day per week had higher prevalence for both overweight and obesity than children involved in school sports more than 2 days per week. The lack of association can partly be attributed to low number of children reported lack of physical activity compared to those reported at least low activity level. The time for waking up in the morning after overnight sleep was not statistically significant factor in this study though results showed children used to wake up very early in between 5:00 am and 6:00 am and children used to wake up at any time in between 5:00 am to 8:00 am (randomly) had higher prevalence for both overweight and obesity than children used to get up in between 6:00 am to 7:00 am (late early). The absence of significance could be due to more participants reporting similar waking up time than other waking up categories. This is supported by the fact that majority of participants were from public day schools and used to walk; therefore, they had to develop a specific timing schedule so that they could get to school early every day.

Concurrent to other reported findings, this study found an association between random sleeping time as compared to normal sleeping time and risk of becoming overweight or obese [41–43]. Participants reported random sleeping time were found to have higher prevalence for both overweight and obesity than participants reported normal sleeping times. This has been attributed to the fact that reducing night sleeping time stimulates overeating [44] by altering levels of hormones involved in hunger and satiety. Random eating of different food categories such as *makande*, pan cake, crisps, juice, and among others without a particular trend of eating a particular food consecutively for at least 5 days per week at school was independent predictor factor for both overweight and obesity. This can be attributed to the fact that this kind of eating habit tends to expose children to food stuff that contain more saturated fats and sugars. Finding like this had also been reported in several studies investigated potential factors for childhood obesity [45–48].

Meal frequency, snacking, eating places, and eating away from home were not statistically significant in this study. Other studies also had reported similar results [49–51]. The lack of association can be attributed to fewer exposures to food opportunities outside homes to the majority of participants. Most children in this study were coming from parents with low socioeconomic status and could afford to attend government day schools. Therefore, majority used to take tea in the morning before going to school and majority used to stay at school for about 8 hrs without eating anything until they went home where used to get lunch and dinner. Not only meal frequency, snacking, eating places, and eating away from home were not statistical but also parental demographic factors. This could be partly attributed to small parents/caregiver sample size due to low response rate.

This is the first study in Arusha City to investigate predictors for overweight and obesity among primary schoolchildren. The uniqueness of this study as compared to previous studies is the investigation of many predictor factors especially that had never been studied in Tanzania. These include sleep timing, time for wake up after overnight sleep, and eating places. The findings are also unique as both obesity and underweight have been observed at the same time. These findings signal an alarm for immediate attention and actions because childhood overweight/obesity and underweight phenomena have been negatively linked with class room performance and achievement [52–54]. Additionally, in adolescents, obesity increases the risks of diseases that were previously seen mostly in adults, including hyperlipidemia, hypertension, metabolic syndrome, and type 2 diabetes [55–57]. Furthermore, it has been reported that obese children tend to become obese adults later at early adulthood [58–60]. Therefore, this is going to inform the government organs responsible for possible causes on the rise of childhood obesity in the country.

This study faced a number of challenges and limitations as follows. Being a town dweller for the participants was most of the challenge that negatively impacted the process of parental data collection [61]. Most of the parents did not feel free to disclose their personal information such as income per month, education level, belongings like owning cars, TV, motor bike, refrigerators, and house, and home eating/dietary habits [62, 63]. This resulted in many incomplete filled parental questionnaires. However, some were so cooperative to provide information about their children and some were nervous in such away they even over reported some information. To address this challenge, sensitization sessions with the parents were first conducted before getting their consenting and participation in the study. This technique worked but at additional cost, and low attendance of participants and prolonged time before data collection could begin were other challenges faced by the researcher. There is therefore a need to educate the public on the need for and importance of research activities to the society and nation at large in order to encourage participation.

## 5. Conclusion

The overall prevalence of overweight and obesity was 17.7% with 12.6% (57/451) being obese and 5.1% being overweight. The prevalence of obesity was as higher as twice the prevalence observed in other previous studies in Tanzania. Being a girl, random sleeping time and random eating habit of different food categories such as *makande*, pan cake, crisps, juice, and among others without a particular trend of eating a particular food consecutively for at least 5 days per week at school were independent predictors of overweight and obesity. However, these findings must be treated with caution because this study was conducted on a small number of schools located in the urban Arusha City over a short period of time. In addition to larger sample sizes, longitudinal studies are needed in order to track individuals and population level trends in BMI over time.

## Figures and Tables

**Table 1:** Participants' sociodemographic characteristics.

Participants' variables	Child sex				Total	
	Girls		Boys			
	*n*	%	*n*	%	*n*	%
*Age groups*						
7–9 years	10	83.3	2	16.7	12	100
10–12 years	116	59.8	78	40.2	194	100
13–15 years	134	55.6	107	44.4	241	100
16–17 years	0	0	4	100	4	100

*Parental income per month*						
Very low income (≤300,000)	47	58.8	33	41.2	80	100
Low income (≤500,000)	42	65.6	22	34.4	64	100
Middle income (≤900,000)	7	43.8	9	56.2	16	100

*Parental occupation*						
Businessman	21	61.8	13	38.2	34	100
Entrepreneur	62	70	27	30	89	100
Mason	1	25	3	75	4	100
Peasant	14	66.7	7	33.3	21	100
Public servant	8	72.7	3	27.3	11	100
Student	0	0	1	100	1	100

*School type*						
Public school	215	62	132	38	347	100
Private school	45	43.3	5	56.7	104	100

*Parental education*						
Tertiary education	4	33.3	8	66.7	12	100
Post-secondary education	1	100	0	0	1	100
Secondary education	9	36	16	64	25	100
Primary	50	41.3	71	58.7	121	100
Not educated	0	0	1	100	1	100

**Table 2:** Overall and child sex mean anthropometric measurements of the study participants.

Variables	Overall (*n* = 451)	Child sex	
		Girls (*n* = 260)	Boys (*n* = 191)
	Mean ± SD	Mean ± SD	Mean ± SD
Age (years)	12.5 ± 1.4	12.4 ± 1.3	12.7 ± 1.4
Weight (kg)	38.1 ± 8.4	38.6 ± 8.5	37.4 ± 8.3
Height (cm)	141.3 ± 18.3	140.1 ± 19.9	142.9 ± 15.7
Waist circumference (cm)	28.4 ± 14.7	27.7 ± 3.0	29.5 ± 22.2
Hip circumference (cm)	32.5 ± 3.4	33.1 ± 3.4	31.7 ± 3.2
Waist-hip ratio	0.9 ± 0.4	0.8 ± 0.1	0.9 ± 0.6
BMI (kg/m^2^)	20.4 ± 9.9	21.5 ± 11.6	18.9 ± 6.7

**Table 3:** Child sex-wise comparison on participants' sociobehaviours.

Participants' variables	Girls		Boys		Total		*X* ^2^	*P* value
	*n*	%	*n*	%	*n*	%		
*Activeness*								
Involved in school sports >4 days/week	5	35.7	9	64.3	14	100	6.924	0.07
Involved in school sports 2–4 days/week	77	61.1	49	38.9	126	100		
Involved in school sports <2 days/week	100	60.3	66	39.7	166	100		
Did not involve in any school sports	23	76.7	7	23.3	30	100		

*Sleeping time*								
10:00 pm and 11:00 pm (late)	72	61	46	39	118	100	0.672	0.87
8:00 pm and 10:00 pm (normal)	101	59.8	68	40.2	169	100		
8:00 pm and 12:00 pm (random)	29	64.4	16	35.6	45	100		
11:00 pm and 12:00 pm (too late)	3	75	1	25	4	100		

*Wake-up timing*								
5:00 am and 6:00 am (early)	171	64.8	93	35.2	264	100	16.01	0.00
6:00 am to 7:00 am (late early)	15	35.7	27	64.3	42	100		
7:00 am and 8:00 am (late)	1	25	3	75	4	100		
5:00 am to 8:00 am (randomly)	18	69.3	8	30.7	26	100		

*Transport*								
On foot	145	61.2	92	38.8	237	100	0.020	0.88
By bus/motor bike	60	60.6	39	39.4	99	100		

*Sedentary life style*								
Spent >3 hrs in TV (high level)	25	47.2	28	52.8	53	100	8.309	0.04
Spent 2-3 hrs in TV (moderate level)	76	65	41	35	117	100		
Spent <2 hrs in TV (low level)	68	58.1	49	41.9	117	100		
Did household chores, no TV (non)	36	73.5	13	26.5	49	100		

Data were presented as proportion and analyzed using chi-square test for comparison.

**Table 4:** Participants' dietary habits and their weight status.

Food items	Under		Norm		Over		Obese		Overall	
	*n*	%	*n*	%	*n*	%	*n*	%	*n*	%
*Snacks*										
Ate highly processed snacks	15	17.9	55	65.5	2	2.4	12	14.2	84	100
Ate less processed snack	29	21.3	78	57.4	7	5.1	22	16.2	136	100
Did not eat snack	9	19.2	33	70.2	1	2.1	4	8.5	47	100
Ate less or highly processed snacks	2	5.7	27	77.1	2	5.7	4	11.5	35	100
Ate sugar-coated snacks	4	11.8	21	61.7	5	14.7	4	11.8	34	100

*Meal frequency*										
Skipped breakfast	12	20.7	36	62.1	4	6.9	6	10.3	58	100
Skipped lunch	7	19.4	23	63.9	3	8.3	3	8.3	36	100
Did not skip meal	40	18.1	134	60.6	10	4.5	37	16.7	221	100

*Eating places*										
Ate at home	32	18.8	99	58.2	9	5.3	30	17.6	170	100
Ate at home or away from home	27	16.4	114	69.1	8	4.8	16	9.7	165	100

*Eating lunch at home*										
Ate lunch meal every day at home	36	18.9	120	63.2	9	4.7	25	13.2	190	100
Ate lunch often away from home	23	15.7	94	64.4	8	5.5	21	14.4	146	100

*School dietary preference*										
Ate fried foods	27	21.8	81	65.3	3	2.4	13	10.5	124	100
Ate refined boiled maize	17	14.9	80	70.2	6	5.3	11	9.6	114	100
Ate nothing	1	20	3	60	0	0	1	20	5	100
Ate any food	11	13.6	44	54.3	8	9.9	18	22.2	81	100
Ate food containing more sugars	3	25	6	50	0	0	3	25	12	100

**Table 5:** Prevalence of overweight and obesity by children sociodemographic characteristics based on univariate logistic regression.

Variables	Overall		Normal		Obese		Overweight		Underweight		*P* value	OR and 95% CI
	*n*	%	*n*	%	*n*	%	*n*	%	*n*	%		
*Child sex*												
Girls	260	100	164	63.1	38	14.6	19	7.3	39	15	0.021	2.06, 1.11–3.82
Boys^*∗*^	191	100	133	69.6	19	9.9	4	2	35	18.3		

*Age groups*												
Below age 10	39	100	30	76.9	5	12.8	3	7.7	1	2.6	0.635	0.82, 0.36–1.85
Above age 10^*∗*^	412	100	267	64.8	52	12.6	20	4.9	73	17.7		

*Activeness*												
Involved in sports >4 days/week	14	100	9	64.3	1	7.1	2	14.3	2	14.3	0.912	1.09, 0.22–5.18
Involved in sports 2–4 days/week	125	100	81	64.8	15	12	5	4	24	19.2	0.599	0.76, 0.27–2.10
Involved in sports <2 days/week	166	100	106	63.8	26	15.7	7	4.2	27	16.3	0.987	0.99, 0.37–2.62
Did not involve in any sports^*∗*^	30	100	18	60	4	13.3	2	6.7	6	20		

*Sleeping time*												
10:00 pm and 11:00 pm (late)	117	100	77	65.8	18	15.4	6	5.1	16	13.7	0.067	1.81, 0.95–3.47
8:00 pm and 10:00 pm (Normal)^*∗*^	169	100	113	66.9	16	9.5	5	2.9	35	20.7		
8:00 pm and 12:00 pm (random)	45	100	21	46.7	12	26.7	4	8.8	8	17.8	0.000	3.88, 1.80–8.35
11:00 pm and 12:00 pm (too late)	4	100	3	75	0	0	1	25	0	0	0.468	2.34, 0.11–19.32

*Wake-up timing*												
5:00 am and 6:00 am (early)^*∗*^	263	100	167	63.5	36	13.7	14	5.3	46	17.5		
6:00 am to 7:00 am (late early)	4	100	3	75	0	0	0	0	1	25	0.985	0.04, 0.13–1.18
7:00 am and 8:00 am (late)	42	100	30	71.4	4	9.5	1	2.4	7	16.7	0.144	0.00, 0.00–1.22
5:00 am to 8:00 am (randomly)	26	100	14	53.8	7	26.9	1	3.8	4	15.4	0.159	1.89, 0.77–4.60

*Transport*												
On foot^*∗*^	236	100	149	63.1	30	12.7	12	5.1	45	19.1		
By bus/motor bike	99	100	65	65.7	16	16.2	4	4	14	14.1	0.605	1.16, 0.64–2.11

*Sedentary*												
Spent >3 hrs in TV (high level)^*∗*^	53	100	35	66	6	11.3	2	3.8	10	18.9		
Spent2-3 hrs in TV (moderate level)	116	100	71	7.1	18	15.5	6	5.1	21	18.1	0.573	1.28, 0.55–3.28
Spent <2 hrs in TV (low level)	118	100	80	67.8	16	13.6	6	5	16	13.6	0.391	1.46, 0.61–3.52
Did house chores, no TV (non)	48	100	28	58.3	6	12.5	2	4.2	12	25	0.829	1.12, 0.38–3.27

^*∗*^Reference groups. OR = odds ratio and CI = confidential intervals.

**Table 6:** Prevalence of overweight and obesity by dietary habits based on univariate logistic regression.

Food items	Overall		Underweight		Normal		Overweight		Obese		*P* value	OR and 95% CI
	*n*	%	*n*	%	*n*	%	*n*	%	*n*	%		
*Meal frequency*												
Skipped lunch^*∗*^	36	100	25	69.4	2	5.6	3	8.3	6	16.7		
No skipped meal	242	100	154	63.6	39	16.1	9	3.7	40	16.5	0.802	1.53, 0.56–4.15
Skipped breakfast	57	100	35	61.4	5	8.8	4	7	13	22.8	0.399	1.16, 0.35–3.79

*Eating places*												
Home^*∗*^	170	100	98	57.7	28	16.5	9	5.3	35	20.5		
Home and away from home	165	100	116	70.3	18	10.9	7	4.2	24	14.5	0.121	0.64, 0.36–1.12

*Lunch at Home*												
Ate lunch every day at home^*∗*^	190	100	120	63.2	24	12.6	8	4.2	38	20		
Ate lunch often away from home	145	100	94	64.8	22	15.2	8	5.5	21	14.5	0.37	1.28, 0.74–2.23

*School dietary preference*												
Ate fried foods	124	100	27	21.8	81	65.3	3	2.4	13	10.5	0.541	0.79, 0.37–1.67
Ate refined boiled maize^*∗*^	114	100	17	14.9	80	70.2	6	5.3	11	9.6		
Ate nothing	5	100	1	20	3	60	0	0	1	20	0.757	1.42, 0.07–10.39
Ate any food	81	100	11	13.6	44	54.3	8	9.9	18	22.2	0.005	2.69, 1.35–5.48
Ate food containing more sugars	12	100	3	25	6	50	0	0	3	25	0.369	1.90, 0.39–7.15

*Snacks eaten at school*												
Yes	288	100	50	17.3	181	62.6	16	5.5	42	14.5	0.141	2.07, 0.78–5.47
No^*∗*^	47	100	9	19.1	33	70.2	1	2.1	4	8.5		

^*∗*^Reference groups. OR = odds ratio and CI = confidential intervals.

**Table 7:** Factors for high BMI percentiles by multiple logistic regression model.

Variables	BMI < 85		BMI > 84		*P* value	AOR and 95% CI
	*n*	%	*n*	%		
*Child sex*						
Girls	203	78.1	57	21.9	0.085	1.77, 0.94–3.45
Boys^*∗*^	168	88	23	12		

*Sleep timing*						
8:00 pm and 10:00 pm (normal)^*∗*^	148	87.6	21	12.4		
8:00 pm and 12:00 pm (random)	29	64.4	16	35.6	0.000	4.47, 2.00–10.01

*School dietary preference*						
Ate refined boiled maize^*∗*^	97	85.1	17	14.9		
Ate any food	55	67.9	26	32	0.012	2.54, 1.23–5.33

^*∗*^Reference groups. AOR = adjusted odds ratio and CI = confidential intervals.

## Data Availability

The data are available from the corresponding author on reasonable request.
